# QTLomics in Soybean: A Way Forward for Translational Genomics and Breeding

**DOI:** 10.3389/fpls.2016.01852

**Published:** 2016-12-21

**Authors:** Giriraj Kumawat, Sanjay Gupta, Milind B. Ratnaparkhe, Shivakumar Maranna, Gyanesh K. Satpute

**Affiliations:** Crop Improvement Section, ICAR—Indian Institute of Soybean ResearchIndore, India

**Keywords:** quantitative traits, genes, alleles, haplotype, functional markers, comparative genomics, legumes

## Abstract

Food legumes play an important role in attaining both food and nutritional security along with sustainable agricultural production for the well-being of humans globally. The various traits of economic importance in legume crops are complex and quantitative in nature, which are governed by quantitative trait loci (QTLs). Mapping of quantitative traits is a tedious and costly process, however, a large number of QTLs has been mapped in soybean for various traits albeit their utilization in breeding programmes is poorly reported. For their effective use in breeding programme it is imperative to narrow down the confidence interval of QTLs, to identify the underlying genes, and most importantly allelic characterization of these genes for identifying superior variants. In the field of functional genomics, especially in the identification and characterization of gene responsible for quantitative traits, soybean is far ahead from other legume crops. The availability of genic information about quantitative traits is more significant because it is easy and effective to identify homologs than identifying shared syntenic regions in other crop species. In soybean, genes underlying QTLs have been identified and functionally characterized for phosphorous efficiency, flowering and maturity, pod dehiscence, hard-seededness, α-Tocopherol content, soybean cyst nematode, sudden death syndrome, and salt tolerance. Candidate genes have also been identified for many other quantitative traits for which functional validation is required. Using the sequence information of identified genes from soybean, comparative genomic analysis of homologs in other legume crops could discover novel structural variants and useful alleles for functional marker development. The functional markers may be very useful for molecular breeding in soybean and harnessing benefit of translational research from soybean to other leguminous crops. Thus, soybean crop can act as a model crop for translational genomics and breeding of quantitative traits in legume crops. In this review, we summarize current status of identification and characterization of genes underlying QTLs for various quantitative traits in soybean and their significance in translational genomics and breeding of other legume crops.

## Introduction

Soybean [*Glycine max* (L.) Merr.] is a major crop used for food, feed, and biofuel on the globe. The soybean has high nutritional value owing to its high protein and oil content, and having health benefits due to presence of isoflavones and tocopherols. The demand for soybean is increasing due to increasing interest in functional food and use of various soybean constituents/by-products in a wide array of specific industrial products. There is a need to develop tailor-made varieties of soybean for specific requirements. While traits like plant architecture, seed yield components, nutrient use efficiency, photosynthetic efficiency, abiotic and biotic stress tolerance are desirable for sustainable higher productivity, traits like protein content, oil content and quality, fragrance, sweetness, tocopherols, and secondary metabolites are desirable for producing good quality consumer products. However, various traits of economic importance in soybean are complex and quantitative in nature therefore difficult to breed for varietal development. The quantitative traits are governed by quantitative trait loci (QTLs). A large number of QTLs have been mapped in soybean for various quantitative traits albeit their utilization in breeding programmes is poorly reported (http://www.soybase.org/). The main reasons for poor utilization of QTLs in soybean breeding programme are; the large size of the mapped genomic regions, small contribution to phenotypic variation, and genetic background effect of recipient parent which demands further validation (Collard and Mackill, 2008; Ratnaparkhe et al., 2014.). For their effective use in breeding programme it is required to narrow down the confidence intervals of QTLs, identify the underlying genes and the most important is allelic characterization of these genes for identifying superior variants.

The soybean genome is complex and probably arisen by two rounds of genome-wide duplications or polyploidy events (Walling et al., 2006; Innes et al., 2008). Although several QTLs were mapped on the soybean genome before the publication of soybean genome sequence however the identification of underlying genes responsible for the quantitative trait of interest was rare (Liu et al., 2008; Watanabe et al., 2009). The availability of genome sequence of soybean has accelerated mapping and identification of genes responsible for various qualitative and quantitative traits (Schmutz et al., 2010). This is further augmented by advancement in high-throughput genotyping and next-generation sequencing (NGS) technologies added with advanced phenotyping facilities. The advantage of gene identification through QTL mapping over several other gene identification approaches is that besides identifying genes directly responsible for pathway enzymes and proteins, trans-acting and regulatory genes can also be identified (gene × gene interaction). Identification of epistatic interactions between QTLs is very important in the development of efficient marker-assisted selection system (Ma et al., 2016).

Comparative genomics is crucial for translating knowledge between model species and crop species, and it is beneficial to consider the legumes as a coherent, broad genetic system (Cannon, 2008). Comparative genomic techniques are useful for positional cloning or finding new genes in related species. Several QTLs in soybean have been characterized for underlying genes identifying causative alleles and associated haplotypes. The knowledge of genetic variants in the form of alleles or haplotypes for underlying genes of QTLs can work as an excellent tool for translational genomics and breeding of quantitative traits in soybean, and other legume crops through comparative genomics approaches. Here, we review current status of identification and characterization of genes underlying QTLs for various quantitative traits in soybean which can facilitate development of tailor-made varieties for trait(s) of interest. While several of the genes underlying QTLs are cloned and functionally characterized, many are identified as candidate genes only and functional validation is required.

## QTLomics: QTLs to genes in soybean

The first report of association of quantitative trait variation with DNA marker loci in soybean was published by Keim et al. (1990a). Since then several advancements in the molecular genetics and genomics had helped in the identification of numerous QTLs in soybean. Advancements from random and limited sequence based marker genotyping to high-throughput sequence based locus-specific marker genotyping lead to mapping of several quantitative traits in soybean (http://www.soybase.org/). The term “QTLomics” deals with the generation and analysis of large scale data-sets for mapping, identification and, structural and functional characterization of the genes underlying quantitative traits. In soybean, QTLomics has been applied for characterization of mapped QTLs as well as for mapping and characterization of new QTLs. Recently, several sequence based data-sets has been generated by re-sequencing efforts to facilitate QTLomics studies in soybean (Table 1; Lam et al., 2010; Li et al., 2013, 2014; Chung et al., 2014; Qiu et al., 2014; Zhou Z. et al., 2015; Valliyodan et al., 2016). The availability of well-annotated soybean genome sequence and re-sequencing based data-sets also facilitate development of large number of SNP and Indel markers which are being utilized in QTL mapping and molecular breeding in soybean. Several SNP based marker assays has been developed and validated in soybean which includes SoySNP50K iSelect BeadChip (Song et al., 2013; Vuong et al., 2015; Zhang et al., 2015a), SoySNP6K Infinium BeadChip (Akond et al., 2013), Axiom SoyaSNP array for ~180,000 SNPs (Lee et al., 2015) and the NJAU 355 K SoySNP array (Wang et al., 2016). Further, recent developments in NGS technologies make sequencing-based genotyping cost-effective and efficient (Deshmukh et al., 2014). These sequencing based genotyping approaches include restriction-site-associated DNA sequencing (Baird et al., 2008; Zhou L. et al., 2015), genotyping by sequencing (GBS) (Elshire et al., 2011; Sonah et al., 2013) and whole genome re-sequencing (Xu et al., 2013; Qi et al., 2014).

**Table 1:** **Whole genome re-sequencing data-sets available for QTLomics in soybean**.

**S. No**.	**No. of accessions used**	**Genotypes**	**Sequencing depth**	**No. of SNPs identified**	**QTL mapping/Putative candidate genes reported**	**References**
1	31	17 *G. soja* and 14 *G. max*	5x	6,318,109	–	Lam et al., 2010
2	25	8 *G. soja* and 17 *G.max* (8 landraces and 9 elite ines/cultivars)	–	5,102,244	Domestication-related traits	Li et al., 2013
3	16	*10 G. max* and 6 *G. soja*	>14x	3,871,469	Domestication-related traits	Chung et al., 2014
4	7	*G. soja*	~111.9x	3.63–4.72M SNP per accession	Biotic resistance, seed composition, flowering and maturity time, organ size, and final biomass	Li et al., 2014
5	11	10 Semi-wild and 1 *G. soja*	9 Semi-wild at ~3X, 1 Semi-wild at ~41X, and 1 Wild at ~55X	7,704,637	Seed size and seed coat color	Qiu et al., 2014
6	302	62 *G. soja*, 240 *G. max* (130 landraces and 110 cultivars)	>11X	9,790,744	Domestication and improvement traits	Zhou Z. et al., 2015
7	106	7 wild, 43 landraces, and 56 elite cultivars	~17X	10,417,285	Oil, protein, salinity, and domestication traits	Valliyodan et al., 2016

Conventionally QTL mapping is performed using bi-parental mapping populations with low density marker coverage. This results in poor resolution of QTLs, identifying large genomic regions with limited information on gene(s) underlying important agronomic traits (Sonah et al., 2015). Genome-wide association study (GWAS), which uses high density marker genotyping in a population of unrelated genotypes that have accumulated a much larger number of crossing-over events since their last common progenitor, provides higher mapping resolution than conventional QTL mapping and enables one to predict or identify causal genes (Zhang et al., 2015a). GWAS has been successfully combined with sequencing based genotyping technologies for identification of QTLs in soybean (Bastien et al., 2014; Sonah et al., 2015; Iquira et al., 2015; Zhou L. et al., 2015) Novel approaches are being applied for QTL mapping and candidate gene identification in legumes which includes use of multi parent populations like nested association mapping (NAM) and multi-parent advanced generation inter-cross (MAGIC) populations, trait mapping through pooled sequencing methods such as QTL-Seq, MutMap, Seq-BSA, Indel-Seq, and BSR-Seq, a comprehensive review of which is given in Pandey et al. (2016). In recent past, several omics resources has been developed in soybean and integration of omics approaches has been proposed for soybean improvement, in which QTLomics will be an integral part (Deshmukh et al., 2014; Chaudhary et al., 2015). Integration of omics approaches leads to QTL mapping and identification of underlying genes, which is the ultimate goal of omics science (Chaudhary et al., 2015).

**Figure 1:**
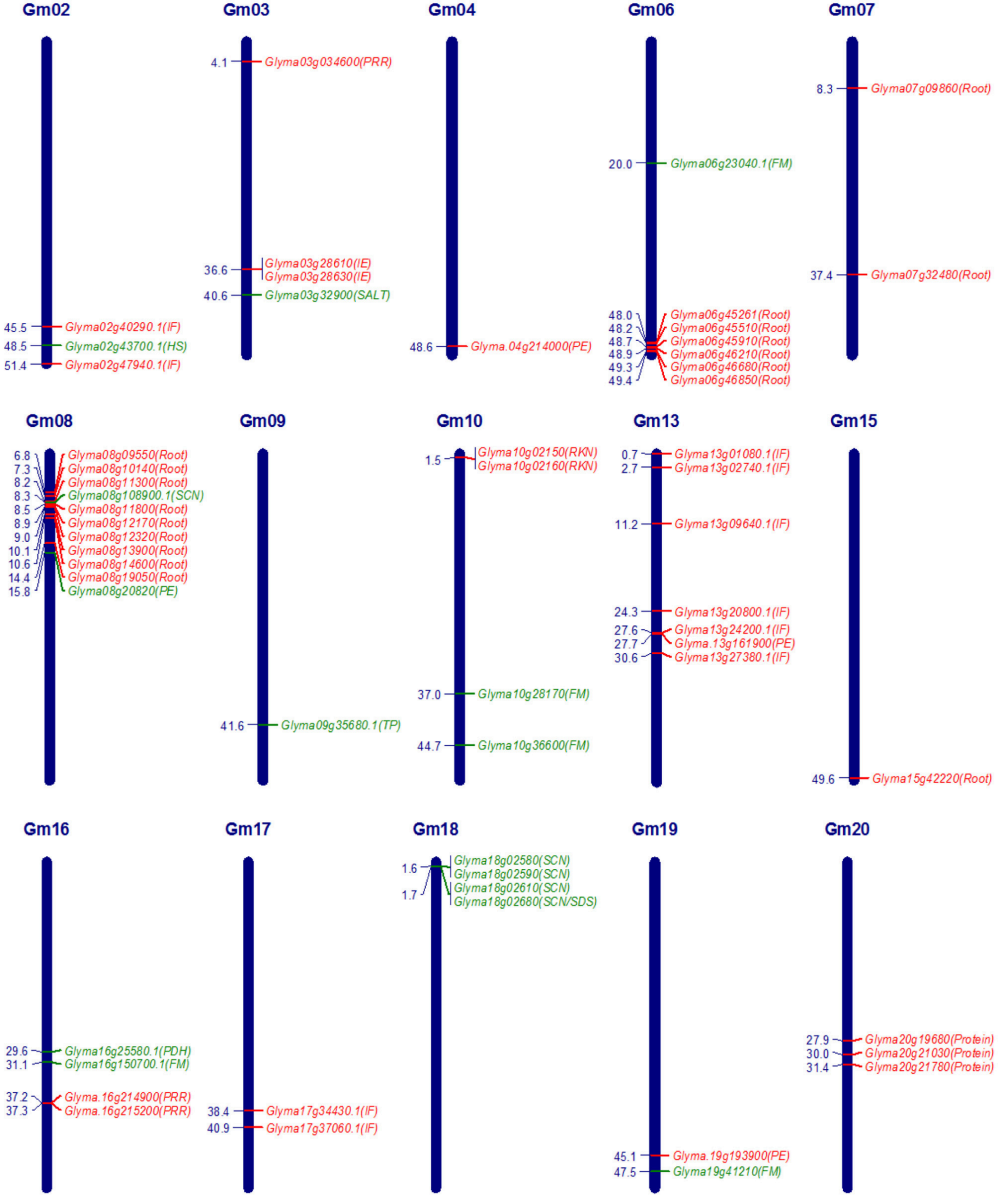
**Map positions (Mbp) of characterized genes and important candidate genes underlying quantitative traits in soybean**. Characterized genes are coded in green colour and important candidate genes are coded in red colour. FM, Flowering and maturity; HS, Hard-seededness; IE, Iron efficiency; IF, Isoflavone content; PE, Phosphorous efficiency; PDH, Pod dehiscence; PRR, Phytophthora root rot; Protein, Protein content; RKN, Root knot nematode; Root, Root traits for drought tolerance; SALT, Salt tolerance; SCN, Soybean cyst nematode; SDS, Sudden death syndrome; TP, α-tocopherol content.

The advancement in the genomic research supported by substantial funding due to commercial importance of soybean crop led to the identification of genes underlying QTLs of several quantitative traits in soybean. Underlying genes identified exclusively through QTL mapping and falling in the confidence interval of QTLs are reported in this review. Putative candidate genes identified in GWAS (Genome-Wide Association Studies) are also included for some of the traits. The identified genes have been divided into two categories, characterized genes and candidate genes. Characterized genes are those which have been functionally validated by gain of function experiments such as genetic transformation, transient gene expression or phenotypic validation in near isogenic lines (NILs), while others identified based on homology and expression analysis were considered as candidate genes for respective QTLs. An overview of characterized genes and important candidate genes in soybean underlying QTLs, for nutrient use efficiency, domestication-related traits, and seed composition traits is given in Table 2, and for biotic and abiotic stress related traits is given in Table 3, which includes information on traits, contrasting parents used in the QTL mapping, gene model number and gene name etc. In soybean, genes underlying QTLs have been identified and functionally characterized for phosphorous efficiency, flowering and maturity, pod dehiscence, hard-seededness, α-Tocopherol content, cyst nematode, sudden death syndrome, and salt tolerance. Whereas, candidate genes underlying QTLs have been identified for iron efficiency, seed weight, protein content, oil content, fragrance, isoflavone content, root knot nematode, bacterial leaf pustule, white mold, *Phytophthora* root rot, and root traits. Map positions of characterized genes and important candidate genes underlying quantitative traits in soybean are depicted in Figure 1. The details of characterized genes, causal genetic variants and functional markers has been presented in this review to facilitate soybean breeders for molecular breeding and genetic manipulation of quantitative traits in soybean.

**Table 2:** **A summary of characterized and candidate genes identified underlying QTLs for nutrient use efficiency, domestication-related, and seed composition traits in soybean**.

**Quantitative trait name**	Contrasting parents used for QTL mapping^*^	**Candidate/characterized gene(s)**	**Gene model number**	**Gene details**	**Method used for functional annotation/characterization**	**References**
**NUTRIENT USE EFFICIENCY**						
Phosphorous efficiency	Nannong 94–156 (Efficient) × Bogao	Characterized	Glyma08g20820	Acid phosphatase (*GmACP1*)	Hairy root transformation	Zhang et al., 2014
	Nannong 94–156 (Efficient) × Bogao	Candidate	Glyma.04g214000	Pectin methylesterase	–	Zhang D. et al., 2016
			Glyma.13g161900	Protein kinase		
			Glyma.19g193900	Purple acid phosphatase		Li H. et al., 2016
Iron efficiency	Clark (Efficient) × PI 54619	Candidate	Glyma03g28610	Basic helix-loop-helix DNA binding domain	RNA-Sequence and NILs	Peiffer et al., 2012
			Glyma03g28630	Basic helix-loop-helix DNA binding domain		
**DOMESTICATION-RELATED TRAITS**						
Flowering and maturity	Misuzudaizu × Moshidou Gong 503 (Early)	Characterized	Glyma06g23040.1	B3 DNA binding protein (*E1*)	Genetic trnsformation	Watanabe et al., 2004; Xia et al., 2012
	Misuzudaizu × Moshidou Gong 503 (Early)	Characterized	Glyma10g36600	GIGANTEA *(GmGIa)*(*E2*)	NILs	Watanabe et al., 2004, Watanabe et al., 2011
	Misuzudaizu × Moshidou Gong 503 (Early)	Characterized	Glyma19g41210	Phytochrome A3 (*GmPhy A3*) (*E3*)	RHLs, NILs	Watanabe et al., 2004, 2009
	Tokei 780 (Early) × Hidaka 4	Characterized	Glyma10g28170	Phytochrome A2 (*GmPhy A2*)(*E4*)	NILs	Abe et al., 2003; Liu et al., 2008
	Tokei 780 × TH85 and TH03 (Early)	Characterized	Glyma16g150700.1	Phosphatidylethanolamine-binding protein (*GmFT2a*) (*E9*)	NILs	Kong et al., 2014; Zhao et al., 2016
Pod dehiscence	Hayahikari (non-shattering) × Toyomusume	Characterized	Glyma16g25580.1	Dirigent-like protein (*GmPdh1*)	Genetic transformation	Funatsuki et al., 2006, 2014
Hard-seededness	Williams 82 × PI 468916 and PI 479752 (Hard-seeded)	Characterized	Glyma02g43700.1	Calcineurin-like metallophosphoesterase transmembrane protein (*GmHs1-1*)	Genetic transformation	Sun et al., 2015
Seed weight	GWAS in 105 wild and 262 cultivated soybean accessions	Candidate	Glyma11g03360	Beta-fructofuranosidase activity	–	Wang et al., 2016
			Glyma11g03430	Oligopeptide transport activity		
			Glyma11g05760	SET domain protein		
			Glyma18g05240	Serine threonine protein kinase		
			Glyma18g43500	Leucine rich repeat protein		
	GWAS in 286 soybean accessions	Candidate	Glyma11g15300	DNA repair protein	–	Zhou L. et al., 2015
			Glyma11g15480	Transcription regulator activity		
			Glyma16g26030	Zinc finger DNA binding protein		
**SEED COMPOSITION TRAITS**						
Protein content	PI468916 (High protein) × A81-356022	Candidate	Glyma20 g18880	Mov34-1 family protein gene	NILs and Microarray	Bolon et al., 2010
			Glyma20 g19680	Heat shock protein (Hsp22.5)		
			Glyma20 g21080	ATP synthase		
		Candidate	Glyma20g18450	Homeobox protein 22 (HB22)	–	Lestari et al., 2013
			Glyma20g18460	Homeobox protein 22 (HB22)		
			Glyma20g18520	Homeobox protein 22 (HB22)		
			Glyma20g18540	Homeobox protein 22 (HB22)		
	GWAS in 106 soybean accessions	Candidate	Glyma20g19680	Heat shock protein	–	Valliyodan et al., 2016
			Glyma20g21030	Ammonium transporter		
			Glyma20g21780	Ethylene receptor		
Fragrance	Kaori (Fragrant) × Chiang Mai 60	Candidate	Glyma05g01770	Betaine aldehyde dehydrogenase 2 (*GmBADH2*)	–	Juwattanasomran et al., 2011
α-Tocopherol content	Keszthelyi Aproszemu Sarga (High) × Ichihime	Characterized	Glyma09g35680.1	γ-tocopherol methyltransferase (γ-*TMT3*)	Heterologous expression in Arabidopsis	Dwiyanti et al., 2011
Isoflavone content	Zhongdou 27 (High) × Jiunong 20	Candidate	Glyma02g40290.1	Cinnamic acid 4-hydroxilase (C4H)	eQTL by Real time-PCR	Wang et al., 2014
			Glyma02g47940.1	Phenylalanine ammonia lyase 1 (PAL1)		
			Glyma17g34430.1	Chalcone isomerase(CHI)		
			Glyma17g37060.1	Dihydroflavonol reductase (DFR)		
			Glyma13g01080.1/2	4 Coumarate: CoA ligase (4CL)		
			Glyma13g02740.1	Flavonol synthase (FLS)		
			Glyma13g09640.1	Chalcone synthase(CHS)		
			Glyma13g24200.1	2-hydroxyisoflavanone synthase (IFS)		
			Glyma13g20800.1	Phenylalanine ammonia lyase 1 (PAL1)		
			Glyma13g27380.1	Dihydroflavonol reductase (DFR)		

* *Beneficial trait of contrasting parent is given in parentheses*.

**Table 3:** **A summary of characterized and candidate genes identified underlying QTLs for biotic and abiotic stress traits in soybean**.

**Quantitative trait name**	Contrasting parents used for QTL mapping^*^	**Candidate/characterized gene(s)**	**Gene model number**	**Gene details**	**Method used for functional annotation/characterization**	**References**
**BIOTIC STRESS**						
Root knot nematode	Magellan × PI 438489B (Resistant)	Candidate	Glyma10g02150 Glyma10g02160	Pectin methylesterase inhibitor (*GmPMI*), Pectin methylesterase inhibitor-pectin methylesterase (*GmPMIPM*)	Quantitative real time-PCR	Xu et al., 2013
Soybean cyst nematode	Essex × Forrest (Resistant)	Characterized	Glyma08g108900.1	Serine hydroxymethyltransferase (*SHMT*)	Virus-induced gene silencing (VIGS), targeted RNA interference (RNAi) and Hairy root transformation	Liu et al., 2012
	Forrest (Resistant)	Characterized	Glyma18g02680	Receptor like kinase *(GmRLK18-1)*	Genetic transformation and NILs	Srour et al., 2012
	PI 88788 (Resistant)	Characterized	Glyma18g02580	Predicted amino acid transporter	RNAi silencing	Cook et al., 2012
			Glyma18g02590	α-soluble N-ethylmaleimide-sensitive factor attachment protein		
			Glyma18g02610	wound-inducible protein 12		
Bacterial leaf pustule	Taekwangkong × Danbaekkong (Resistant)	Candidate	Glyma17g09780	Unknown-membrane protein	–	Kim D. et al. (2010)
			Glyma17g09790	Zinc finger protein		
Soybean white mold	Maple Arrow (Partially resistant) × Hefeng 25	Candidate	Glyma13 g03360	PR5-like receptor kinase	–	Zhao et al., 2015
			Glyma13 g04020	RINT-1/TIP-1 family		
			Glyma13 g04031	Myb domain protein 33		
			Glyma13 g04041	C2H2-type zinc finger family protein		
Sudden death syndrome	Essex × Forrest (Resistant)	Characterized	Glyma18g02680	Receptor like kinase *(GmRLK18-1)*	Genetic transformation and NILs	Srour et al., 2012
	GWAS in 214 soybean accessions	Candidate	Glyma02g11270	Stress induced receptor-like kinase 1 (*GmSIK1*)	–	Zhang et al., 2015b
*Phytophthora* root rot	GWAS in 279 soybean accessions	Candidate	Glyma13g32980	*Coat protein I* (COPI)-related gene	–	Li et al., 2016b
			Glyma13g33900 Glyma13g33512	2OG-FE(II) oxygenase family protein Pentatricopeptide (PPR) repeat-containing protein		
			Glyma13g33536	Leucine-rich repeat domain protein		
			Glyma13g33740	Leucine-rich repeat domain protein		
			Glyma13g33243	Gpi16 subunit		
			Glyma13g33260	Zn-finger protein		
	Williams × PI 567139B (Resistant)	Candidate	Glyma.03g034600	AAA-type ATPase family protein	RNA-seq analysis	Li et al., 2016a
			Glyma.16g215200	TIR-NBS class disease resistance protein		
			Glyma.16g214900	TIR-NBS-LRR class disease resistance protein		
**ABIOTIC STRESS**						
Root traits for drought tolerance	V71-370 (High) × PI 407162	Candidate	Glyma06g45510	*D6 type cyclin* gene	Micro-array analysis	Prince et al., 2015
			Glyma06g45261	Auxin efflux carrier protein		
			Glyma06g46680	Alpha/beta hydrolase		
			Glyma06g46850	Histone-like CCAAT transcription factor		
			Glyma06g46210	NEDD8-activating complex		
			Glyma06g45910	Peroxidise 3		
			Glyma09g32280	Kinesin motor family protein		
			Glyma08g19050	Metacaspase-1		
			Glyma07g09860	Triglyceride lipase		
			Glyma07g32480	Apoptosis inhibitory 5 family protein		
			Glyma15g42220	Slow anion channel associated 1-like		
	Dunbar (High) × PI 326582A	Candidate	Glyma08g12320	MYB-HD transcription factor	RNA-seq and Affymetrix expression data	Manavalan et al., 2015
			Glyma08g09550	TPR transcription factor		
			Glyma08g11800	C2H2 Zn transcription factor		
			Glyma08g12170	bZIP transcription factor		
			Glyma08g10140	GRAS transcription factor		
			Glyma08g13900	Ring finger transcription factor		
			Glyma08g14600	AP2- EREBP transcription factor		
			Glyma08g11300	Xyloglucan endo-transglycosylases (*XET*)		
Salt tolerance	W05 (Tolerant) × C08	Characterized	Glysoja01g005509/Glyma03g32900	*Cation/proton exchanger family* gene (*GmCHX1*)	Hairy root transformation	Qi et al., 2014
					Transgenic Tobacco BY-2 cells	
	Tiefeng 8 (Tolerant) × 85–140	Characterized	Glyma03g32900.1	*Cation/proton exchanger family* gene (*GmSALT3*)	NILs grafting	Guan et al., 2014b
	FT-Abyara (Tolerant) × C01	Characterized	Glyma03g32900	*Cation/proton exchanger family* gene (*Ncl*)	Genetic transformation and NILs grafting	Do et al., 2016

* *Beneficial trait of contrasting parent is given in parentheses*.

### Genes for nutrient use efficiency traits

#### Phosphorous efficiency

Phosphorus (P) is the second most important plant nutrient available in soil after nitrogen. Though soils usually contain high amount of total phosphorous, most of the phosphorous (50–80%) occur in organic form which is unavailable to plants. In response to persistent P deficiency conditions, plants have developed many adaptive mechanisms which enhance the availability and uptake of P (Jain et al., 2007). One of the strategies that plants adapt to overcome P deficiency is to secrete acid phosphatases for the hydrolysis of these organic phosphates and thereby using the released inorganic phosphates (Wang et al., 2009). Activity of acid phosphatases (APases) enhances significantly with phosphate deficiency or starvation (Kummerova, 1986). Wang et al. (2009) showed that the over-expression of Arabidopsis *acid phosphatase* gene (*AtPAP15*) in soybean significantly enhanced the APase activity that results in improved P efficiency and plant dry weight as compared with the wild type plants. In Soybean, Zhang et al. (2014) identified a highly significant QTL *qPE8*, on Gm08, in a recombinant inbred lines (RIL) population derived from a cross between the “Nannong 94–156” variety that possessed high P efficiency and the “Bogao” variety with low P efficiency. The *qPE8* was delineated in a genomic region of ~250 kb by using regional association mapping. The delineated genomic region contained a potential candidate gene *Glyma08g20820*, encoding an acid phosphatase (*GmACP1*). *GmACP1* over-expression in soybean hairy roots exhibited a 2.3 fold increase in APase activity and 11–20% increase in P efficiency relative to the control (Zhang et al., 2014). The natural polymorphism analysis of *GmACP1* in 192 soybean accessions identified six probable causative sites that were significantly associated with variations in P efficiency related traits. Ten haplotypes classes were identified for these six sites that explained 33% of the phenotypic variation in P efficiency (Zhang et al., 2014). The cloning of *GmACP1* gene, identification of different haplotypes with their phenotypic effects and availability of functional markers will help breeders for improving P efficiency in soybean. Moreover, the identification of optimal haplotype of *GmACP1* among soybean germplasm collections could further improve the efficacy of selecting soybeans genotypes with higher P efficiencies.

More recently, a high-density genetic map of the same RIL population identified a novel and highly significant QTL *q4-2*, located on Gm04 (Zhang D. et al., 2016). Out of the 38 annotated genes in this QTL region, a *pectin methylesterase* gene and a *protein kinase* gene were identified as promising candidates involved in regulating soybean P efficiency. To elucidate the genetic relationship between phosphorus efficiency and photosynthetic traits, Li H. et al. (2016) identified three major QTLs, *q14-2, q15-2*, and *q19-2*, which were found to be associated with both the traits. Within the *q19-2* region, a *purple acid phosphatase* gene (*Glyma.19G193900*), showing significant differential expression upon low P stress was identified as a promising candidate gene involved in regulating both soybean P efficiency and photosynthetic capacity (Li H. et al., 2016).

#### Iron efficiency

Like phosphorus, soils are abundant in iron but most of it is present in the form of Fe^3+^ which is not accessible to plants. The Fe^2+^ form is physiologically more significant for plants but it readily oxidizes to sparingly accessible form (Fe^3+^). Fe^3+^ is insoluble in neutral and high pH, making iron unavailable to plants in alkaline and in calcareous soils. Furthermore, in these types of soil, iron readily combines with phosphates, carbonates, calcium, magnesium, and hydroxide ions and results in poor iron availability to plants. These conditions cause iron deficiency chlorosis (IDC) in soybean, in which photosynthetic pigments, electron transport potential, and thylakoid system are reduced as plants fail to utilize iron from the soil (Spiller and Terry, 1980; Taylor et al., 1982). The best way to manage IDC is the use of iron-efficient varieties in commercial crops (Peiffer et al., 2012). Lin et al. (1997) identified a major QTL for iron efficiency on Gm03, explaining more than 70% of the phenotypic variation in the Anoka × A7 mapping population. Using introgression mapping, transcriptome sequencing and gene expression analysis, Peiffer et al. (2012) characterized this QTL and identified two candidate genes *Glyma03g28610* and *Glyma03g28630*, encoding transcription factors. These two transcription factor encoding genes, were identified as homologs of the subgroup *Ib basic helix-loop-helix (bHLH)* genes that are known to regulate the strategy I response adaptation in Arabidopsis (Wang et al., 2007; Peiffer et al., 2012). These two genes were significantly induced in soybean roots under iron limiting conditions. Re-sequencing of these two induced genes identified a significant deletion of 12-bp within a predicted dimerization domain of *Glyma03g28610* in iron-inefficient lines (Peiffer et al., 2012). Further experiments for functional validation and identifying the target genes for DNA binding of the two *basic helix-loop-helix (bHLH)* genes may help in identification of iron efficiency molecular network in soybean.

### Genes for domestication-related traits

#### Flowering and maturity

Time to flower and mature confer adaptation of a crop to a specific geographical area. Soybean is a quantitative photoperiod-sensitive short-day plant and its individual genotypes start flowering when the day length becomes less than their critical day length. Ten maturity loci, designated as *E* loci, have been characterized by classical methods in soybean. These loci are *E1* and *E2* (Bernard, 1971), *E3* (Buzzell, 1971), *E4* (Buzzell and Voldeng, 1980), *E5* (McBlain and Bernard, 1987), *E6* (Bonato and Vello, 1999), *E7* (Cober and Voldeng, 2001), *E8* (Cober et al., 2010), *E9* (Kong et al., 2014), and *J* (Ray et al., 1995). Of these, *E1, E3, E4*, and *E7* have been reported as photoperiodic loci (Cober and Voldeng, 2001). Dominant alleles at *E6, E9*, and *J* promote early flowering, whereas dominant alleles at other loci delay flowering and maturity (Zhao et al., 2016). *E1, E2, E3, E4*, and *E9* loci were mapped as QTLs and underlying genes were identified as a B3 DNA binding protein, *GIGANTEA, Phytochrome A3, Phytochrome A2*, and *GmFT2a*, respectively (Liu et al., 2008; Watanabe et al., 2009, 2011; Xia et al., 2012; Zhao et al., 2016). Molecular bases of *E5, E6, E7, E8*, and *J* are unknown.

Out of the known *E* loci in soybean, the *E1* locus is considered to have the largest effect on time to flowering under field conditions (Stewart et al., 2003). Xia et al. (2012) resolved this QTL into a single gene that encodes a legume specific transcription factor containing B3 domain with a putative bipartite nuclear localization signal (NLS) and C-terminal region carrying structure for DNA binding. Three mutations, a hypoactive allele *e1-as*, and two dysfunctional alleles *e1-nl* and *e1-fs* have been reported at this locus by Xia et al. (2012). A new allele with an insertion of a long interspersed nuclear element (LINE) at the promoter of the *E1* locus (*e1-re*) was identified in “Gokuwase-Kamishunbetsu” (Tsubokura et al., 2014). Soybean maturity locus *E2* has been identified as *GIGANTEA* (*GmGIa*), an ortholog of *Arabidopsis thaliana GIGANTEA* (*GI*) gene (Watanabe et al., 2011). A single nucleotide polymorphism (SNP) identified in the 10th exon was responsible for early flowering in Misuzudaizu (Watanabe et al., 2011). Two new alleles with insertion and deletion of 36 bases in the eighth intron (*E2-in* and *E2-dl*) were identified at the *E2* locus by Tsubokura et al. (2014). Since the expression of *GI* gene is not influenced by photoperiod, null mutations in the *GI* gene may be useful resources for adapting plants with complex genomes, such as soybean, to a wide range of geographic regions (Watanabe et al., 2011).

Yamanaka et al. (2001) identified QTL *FT3* and suggested that it may be identical to *E3* locus. Using map-based cloning strategy, this QTL has now been resolved as a phytochrome gene *GmPhyA3* (Watanabe et al., 2009). One SNP for a nonsynonymous amino acid substitution in the third exon and a large deletion of 13.33 kbp at a position after the third exon were detected as two different dysfunctional alleles of *GmPhyA3* (Watanabe et al., 2009). Three novel dysfunctional alleles, *e3-ns* in exon 3 and *e3-fs* in exon 1 and *e3-tr* in exon 4 have been reported by Xu et al. (2013). Using candidate gene approach, Liu et al. (2008) identified E4 as *GmPhyA2*, a homolog of *phytochrome A*. At recessive *E4* locus, a *Ty1/copia*-like retrotransposon was inserted in the exon 1 of the *GmphyA2* gene, which resulted in dysfunction of this gene (Liu et al., 2008). At least five dysfunctional alleles are known in *GmphyA2* gene (Tsubokura et al., 2013). Loss-of-function alleles at *GmPhyA3* and *GmphyA2* can result in some degree of suppression of *E1* transcription and correspondingly elevate *GmFT* expression, leading to relatively early flowering (Xia et al., 2012).

The *E9* locus was mapped by Kong et al. (2014) using two backcross derived populations developed from Tokei 780 and two of the RILs derived from the cross Tokei 780 × Hidaka 4 (a wild soybean accession). Fine mapping of the QTL region identified two candidate genes *GmFT2a* and *GmFT2b* for early flowering (Kong et al., 2014). Zhao et al. (2016) resolved this QTL as leaky allele of *GmFT2a*. The *e9* allele had an insertion of 6.22 kb *Ty1/copia*–like retrotransposon, *SORE-1*, in the first intron which attenuated *FT2a* expression by its allele-specific transcriptional repression (Zhao et al., 2016). The maturity genes *B3 DNA binding protein* gene (*E1*), *GIGANTEA* (*E2*), *GmPhyA3* (*E3*), and *GmPhyA2* (*E4*) down regulate expression of *GmFT2a* (*E9*) and *GmFT5a* to delay flowering and maturation under the long day condition, suggesting that *GmFT2a* and *GmFT5a* are the major targets in the control of flowering in soybean (Kong et al., 2010; Thakare et al., 2011; Watanabe et al., 2011; Xia et al., 2012; Zhao et al., 2016). The genes and alleles identified for flowering and maturity traits in soybean have been validated in several studies using germplasm from many countries (Tsubokura et al., 2013, 2014; Jiang et al., 2014; Langewisch et al., 2014; Zhai et al., 2014; Gupta et al., 2016). A lot of information has been generated on flowering and maturity genes in soybean which also reveals the presence of legume specific unique genes in soybean i.e., *B3 DNA binding protein* gene of *E1* loci. This information is very useful for identification of genes and their haplotypes in other legume species, which can be used for development of geographical region specific suitable genotypes through molecular breeding.

#### Pod dehiscence

Pod dehiscence is a critical step in the seed dispersal of legumes and especially in wild species of crop plants. However, it can cause significant yield losses in soybean and shattering resistant genotype is desirable. The cultivated soybean has been domesticated from its annual wild relative, *Glycine soja* (Sieb et Zucc), in China ~5000 years ago (Hymowitz and Singh, 1987) resulting in a multitude of soybean landraces that have adapted to various climate environments. Although, the cultivated soybean is more shattering resistant than the wild soybean, genetic variation in the degree of pod dehiscence is also present among cultivated soybean (Tsuchiya, 1987). Bailey et al. (1997) identified a major QTL *qPdh1* on linkage group J, with a phenotypic variance contribution of 50% for pod dehiscence in cultivated soybean. QTL *qPdh1* was later confirmed in other studies and fine-mapped to a 47-kb genomic region (Funatsuki et al., 2006, 2008; Suzuki et al., 2010; Gao and Zhu, 2013). Using fine mapping and complementation test, Funatsuki et al. (2014) identified an underlying gene *Glyma16g25580.1* (*Pdh1*), which encodes a dirigent-like protein known to be involved in the lignifications which increases dehiscing forces by increasing torsion of dried pod walls. In the shattering-resistant genotype Hayahikari, the gene *pdh1* was defective, having a premature stop codon due to the presence of a non-sense SNP in the coding sequence (Funatsuki et al., 2014). *Pdh1* is a novel gene identified for pod dehiscence and selection of nonsense mutation in this gene has played an important role in soybean domestication. Functional markers developed based on this nonsense SNP could be utilized for development of shattering resistant soybean genotypes through marker-assisted selection (MAS).

#### Hard-seededness

Wild species of leguminous crops produces seeds with variable seed-coat impermeability as a mechanism for maintaining seed viability for long periods (Fuller and Allaby, 2009), which is essential for the long-term survival of wild species (Foley, 2001). A moderate level of seed coat impermeability or hard-seededness is important for the stored soybean seeds as it is desirable for their quality and viability in the tropics, where seeds lose viability within a short period of time after being harvested (Potts et al., 1978). Several studies have identified a common QTL underlying hard-seededness to an overlapping region on soybean Gm02 (Keim et al., 1990b; Watanabe et al., 2004; Liu et al., 2007; Zhang et al., 2008), which has been further confirmed by Sun et al. (2015) and designated as *(GmHs1-1)*. Sun et al. (2015) could delimit this QTL to a 22-kb region which contained only two genes, *Glyma02g43700.1* and *Glyma02g43710.1*. Sequence characterization of these two genes identified a single SNP (C>T) in 8th exon of *Glyma02g43700.1* which was responsible for the change of threonine amino acid to methionine. This SNP could distinguish seed coat permeable parent from two hard seed coat *G. soja* parents and 8 additional *G. soja* accessions. Genetic transformation of permeable seed coat accession with *Glyma02g43700.1* and observed phenotypic segregation confirmed that *Glyma02g43700.1* is *GmHs1-1* locus (Sun et al., 2015). *Glyma02g43700.1* was predicted to encode a calcineurin-like metallophosphoesterase transmembrane protein that localized to cellular membranes and its transcript were found predominantly in the Malpighian layer of the seed coat (Sun et al., 2015). These workers also reported that in addition to selection for this major mutation in *GmHs1-1*, there have been selections against many minor QTLs of *G. soja* during domestication for the development of permeable seed coat in *G. max*.

#### Seed weight

Seed weight is another very important domestication trait which is under continuous selection pressure. Wild species of soybean *G. soja* has very small seeds and selection for bold seeded lines during domestication has given commercial value to this crop. Using GWAS in 286 soybean accessions, Zhou L. et al. (2015) identified two candidate genes on Gm011 and three candidate genes on Gm16 significantly associated with seed size. Three of the five candidate genes had shown relatively higher expression levels during seed development. One candidate gene *Glyma11g15480* was homologous to the *Arabidopsis* gene *NOT2A* (Wang et al., 2013). A correlation analysis based on the gene expression dataset showed that *Glyma11g15480* is co-expressed with 24 genes homologous to seed development genes in Arabidopsis (Zhou L. et al., 2015). In another GWAS experiment using 367 accession of wild and cultivated soybean, Wang et al. (2016) reported two candidate genes for seed weight on Gm11 in cultivated soybean and three candidate genes on Gm11 and Gm18 in wild soybean gene-pool. Among the two candidate genes identified in cultivated soybean, *Glyma11g03360* is homologous to *Os04g33740* (*GIF1*) that regulates grain filling and size in rice, and *Glyma11g03430* is homologous to O*s11g12740* (*sp1*), a gene involved in panicle elongation and grain size (Wang et al., 2008; Li et al., 2009).

### Genes for seed composition

#### Protein content

Soybean is a rich source of protein therefore it is imperative to investigate genes responsible for seed protein accumulation. β-conglycinin and glycinin are the most prominent seed proteins in soybean (Roberts and Briggs, 1965; Thanh and Shibasaki, 1976). Several seed protein QTLs have been identified in soybean and among them QTLs of LG I (Gm20) are important as they have been reported in many of the mapping populations (Diers et al., 1992; Seboldt et al., 2000; Csanádi et al., 2001). Bolon et al. (2010) could also identify QTL for seed protein at this linkage group and characterized the underlying genes by using a near-isogenic line (NIL) pair that contrasted in seed protein content. This NIL pair differed in protein content because of introgression of a genomic segment containing the high protein QTL of *G. soja* (PI468916) into *G. max* A81-356022. Using microarray and high-throughput whole transcriptome sequencing approaches, 13 genes displaying significant seed transcript expression between NILs were identified which included a potential regulatory protein of the Mov34-1 family, a heat shock protein Hsp22.5, and an ATP synthase (Bolon et al., 2010). In a regional association study of genomic block on Gm20, Valliyodan et al. (2016) identified that the copy number variations in candidate genes (HSP, *Glyma20g19680*; ammonium transporter, *Glyma20g21030*; ethylene receptor, *Glyma20g21780*) were associated with protein content. Lestari et al. (2013) reported that parts of Gm20 and Gm10 showed synteny, however QTLs for seed protein content were detected only on Gm20. Comparative sequence analysis identified that four tandem duplicates of the putative homeobox protein 22 (HB22) were present specifically on Gm20 (Lestari et al., 2013). This *Medicago truncatula* homolog has been reported to be expressed in endosperm and hypothesized to regulate seed filling stage (Verdier et al., 2008). Like *Medicago truncatula*, these tandem duplicates could be regulating seed filling stage in soybean as well and therefore contribute to the seed protein QTL of Gm20. Further, functional studies are required to identify and validate the role of these candidate genes in seed protein accumulation.

#### Fragrance

One of the food uses of soybean is the young pods harvested at R6 stage, cooked and consumed as snack or vegetable (Shanmugasundaram et al., 1991). Soybean varieties used for this purpose are called as “vegetable or edamame or green soybean” and they have some unique characteristics like highly sweet seeds, extra large pods and seeds, and distinct seed fragrance. The vegetable soybean cultivars “Dadachamame” and “Chakaori” are reported to have pleasant fragrance due to the presence of a volatile compound 2-acetyl-1-pyrroline (2AP) (Fushimi and Masuda, 2001), which is also found in fragrant rice. A single recessive gene has been reported to control the fragrance in vegetable soybean (AVRDC, 2003). In rice, recessive mutations in the *betaine aldehyde degydrogenase 2* gene (*OsBADH2*) are responsible for fragrance (Bradbury et al., 2005; Wanchana, 2005; Shi et al., 2008). This recessive mutation causes elevated 2AP biosynthesis that result in a fragrant aroma in those varieties (Niu et al., 2008; Vanavichit et al., 2008). Through *in-silico* study and QTL analysis Juwattanasomran et al. (2011) identified a major QTL for fragrance in soybean which coincides with the position of *betaine aldehyde dehydrogenase 2* (*GmBADH2*), the same gene responsible for the fragrance in rice (Bradbury et al., 2005). Sequence comparison of *GmBADH2* between Kaori (Fragrant) and non-fragrant soybeans revealed a non-synonymous SNP in exon 10 resulting in change of glycine to aspartic acid. Five PCR-based allele specific SNP markers were developed for marker-assisted breeding of fragrance trait in soybean (Juwattanasomran et al., 2011). A new fragrance allele, which has a 2-bp (TT) deletion in exon 10 of *GmBADH2*, was discovered in another fragrant soybean cultivar Chamame by Juwattanasomran et al. (2012) and functional markers have been developed for MAS.

#### α-tocopherol content

Tocopherols contribute to both; the nutritional value of soybean seeds and the oxidative stability of soybean oil. The major forms of tocopherols found in seed of common soybean cultivars are γ-tocopherol and δ-tocopherol, which account for 60–70 and 20–25% of the total tocopherol, respectively. The proportion of α-tocopherol, which is having highest vitamin E activity in mammals, is usually <10% of total tocopherol in soybean seeds (Van Eenennaam et al., 2003; Ujiie et al., 2005). Owing to its antioxidant activity, Vitamin E is widely used in food and oil. It's also used as a supplement in the human diet to reduce the risk of cancer and cardiovascular diseases and as a nutrient additive in poultry and cattle feed to improve meat quality. Dwiyanti et al. (2011) identified gene underlying QTL for high α-tocopherol content in a soybean variety Keszthelyi Aproszemu Sarga (KAS). Through QTL analysis and fine mapping in populations from a cross between KAS and a Japanese variety Ichihime, they identified γ*-TMT3*, which encodes γ-tocopherol methyltransferase, as a candidate gene responsible for high α-tocopherol concentration in KAS. Expression analysis and genetic transformation of γ*-TMT3* promoter from KAS in transgenic Arabidopsis plants identified transcriptional regulation of γ*-TMT3* (Dwiyanti et al., 2011).

#### Isoflavone content

Isoflavones are health beneficial molecules in human nutrition (Tsukamoto et al., 1995; Rochfort and Panozzo, 2007). In soybean, isoflavones are critical factor in defense against pathogens (Benhamou and Nicole, 1999; Subramanian et al., 2004), in promoting nodulation by rhizobia (Subramanian et al., 2006, 2007) and in changing or adjusting the microbial population around plant roots (Lozovaya et al., 2004). Several QTLs have been mapped for three species of isoflavones i.e., genistein (*GEN*), daidzein (*DAI*), glycitein (*GLY*), and total of isoflavones (*TOT*) (Primomo et al., 2005; Zeng et al., 2009; Gutierrez-Gonzalez et al., 2010, 2011; Wang et al., 2014). Gutierrez-Gonzalez et al. (2010, 2011) identified and validated a major QTL in two RILs derived from low isoflavone lines Magellan and Essex, and high isoflavone line PI 437654. This QTL was detected on Gm05 in both of the studies and had significant effect on individual isoflavone and total seed isoflavone accumulation (named *qGEN5, qDAI5, qGLY5*, and *qTOT5*, respectively). This locus accounted for about 36, 35, 12, and 37% of the variance for genistein, daidzein, glycitein, and total isoflavone concentration, respectively. Another major QTL has been identified on Gm08 by Yang et al. (2011). Gutierrez-Gonzalez et al. (2010) used candidate gene approach to identify homologous sequences in the soybean genome with assigned or putative functions in tocopherol biosynthesis pathway, and placed them on the map at their approximate positions, along with all reported QTLs using their interval of confidence. The study identified number of putative candidate genes underlying several of the main-effect and epistatic QTLs. Since there is possibility of trans-acting regulatory elements responsible for the phenotype, Wang et al. (2014) used expression QTL (eQTL) analysis combined with phenotypic QTL analyses identifying a total of 33 eQTLs, of them five eQTL intervals were overlapped with phenotypic QTLs (pQTLs). A total of 11 candidate genes were identified within the overlapped eQTL and pQTL genomic regions (Table 2; Wang et al., 2014). Identification of causative genetic variants within these genes and structural variations in flanking regions is required for development of haplotype based molecular markers facilitating manipulation of isoflavone content in soybean.

#### Oil content

Oil content of soybean is a valuable quantitative trait controlled by multiple genes. At least 110 QTLs for soybean oil content have been mapped but the utilization of these QTLs for soybean breeding has some limitations. Recently, Valliyodan et al. (2016) conducted comparative sequence analysis between high and low oil lines and showed differential variation in SNP and copy number for lipid biosynthesis and lipid degradation genes between high oil and low oil lines. The comparative analysis shows increased copy number of lipid transfer protein (LPT; *Glyma16g31780, Glyma16g31840*, and *Glyma16g31540*) in high oil lines, whereas the negative regulators of lipid biosynthesis genes (ABC transporter, *Glyma03g36310*, Lipase 3, and *Glyma13g04561*) showed more copies in low oil lines. The differential variation for lipid biosynthesis and lipid degradation genes between high oil and low oil lines might be associated with the differences in oil content.

### Genes for biotic stress

#### Nematode resistance

##### Root knot nematode

Nematode causes severe yield losses in soybean in the regions of warm climate and sandy soils (Walters and Baker, 1994). The root not nematode and soybean cyst nematode are two major pests of soybean in United States. Using whole genome re-sequencing of recombinant inbred lines (RIL) derived from a cross between Magellan (susceptible) and PI 438489B (resistant), Xu et al. (2013) identified two candidate genes underlying a major QTL for root not nematode resistance mapped to bin 10 of Gm10. The two candidate genes *Glyma10g02150* and *Glyma10g02160*, encodes a pectin methylesterase inhibitor and a pectin methylesterase inhibitor-pectin methylesterase, respectively (Xu et al., 2013). The protein encoded by *Glyma10g02160* was found similar to the *Arabidopsis* protein pectin methylesterase, PME3, which was shown to be important in nematode parasitism (Hewezi et al., 2008). Although, SNPs and InDels were reported between parental lines for both genes but functional characterization and haplotype analysis is required for marker development and utilization.

##### Soybean cyst nematode

Soybean cyst nematode (SCN) (*Heterodera glycines* Ichinohe) feeds on the roots of soybean and is a major constraint to soybean production in United States. In resistant genotype Peking, a dominant resistance gene (*Rhg4*) was identified that was linked to *I* locus underlying seed coat color (Matson and Williams, 1965). Through positional cloning from soybean cultivar Forrest, Liu et al. (2012) identified a gene underlying *Rhg4* locus on Gm08, a major QTL contributing to resistance to SCN. The gene underlying *Rhg4* encodes a serine hydroxymethyltransferase (SHMT) enzyme, a ubiquitous enzyme in nature with a key role in one-carbon folate metabolism. Alternative alleles of *Rhg4* differ by two genetic polymorphisms that alter a key regulatory property of the enzyme (Liu et al., 2012). Haplotype survey of *SHMT* sequence identified eight haplotypes, where soybean lines with haplotypes H1–H3 carry resistant alleles at *SHMT* (Liu et al., 2012). The identified resistance mechanism can be readily exploited to improve resistance against SCN in susceptible cultivars. A recessive and codominant locus *rhg1* which is necessary, but not sufficient, for resistance to all known Hg-biotypes of SCN, was also mapped (Concibido et al., 1997; Ruben et al., 2006). Ruben et al. (2006) fine mapped the *rhg1* locus and identified a single *receptor like kinase* (*RLK; leucine rich repeat transmembrane-protein kinase*) as candidate resistance gene. Srour et al. (2012) characterized the *GmRLK18-1*, the gene underlying *Rhg1* locus, in transgenic and heterozygous plants and demonstrated that the dominant allele confers reported pleiotropic resistance to SCN and sudden death syndrome (SDS) caused by *Fusarium virguliforme*. In the absence of *Rhg4*, the *GmRLK18-1* had provided partial resistance to SCN and conferred nearly complete resistance to SDS, whereas, in the presence of *Rhg4*, the plants with the *GmRLK18-1* transgene were fully resistant to SCN as well as SDS (Srour et al., 2012). *In vitro*, the purified LRR domain of *GmRLK18-1* has been shown to bind with the CLE peptides of plant origin known to involve in tracheary element inhibition (Afzal et al., 2013).

About 90% of the commercially cultivated SCN-resistant soybean varieties in the central United States use the *rhg1-b* allele (haplotype) as the main SCN resistance locus derived from the genotype PI 88788 (Cook et al., 2012). Fine mapping of *rhg1-b* haplotype in PI88788 has delineated *rhg1-b* in an interval that corresponds to a 67-kb segment carrying 11 predicted genes in the genome of Williams 82 (Kim M. et al., 2010). By using gene silencing approach, Cook et al. (2012) identified that SCN resistance mediated by the soybean QTL *Rhg1* in PI 88788 is conferred by copy number variation of a 31-kilobase segment encoding an amino acid transporter (*Glyma18g02580*), an α-SNAP (α-soluble N-ethylmaleimide-sensitive factor attachment protein) protein (*Glyma18g02590*), and a WI12 (wound-inducible domain) protein (*Glyma18g02610*). Further, characterization of genetic variation at *Rhg1* across 41 diverse soybean accessions discovered that SCN resistance is associated with multicopy *Rhg1* haplotypes that form two distinct groups (Cook et al., 2014). The high-copy-number *Rhg1* accessions reportedly contain a flexible number of copies (seven to 10) of the 31-kb *Rhg1* repeat whereas the low-copy-number *Rhg1* group contains three copies of the *Rhg1* repeat and a newly identified allele of α-SNAP (Cook et al., 2014). Kadam et al. (2016) selected SNPs from the *Rhg1* gene (*Glyma18g02590*) and *Rhg4* gene (*Glyma08g11490*) to develop KASPar assays. Two SNP markers specific to *Rhg1* locus and two SNP markers specific to *Rhg4* locus were validated in 95 germplasm lines and a subset of RILs. A Taqman assay was also developed from the conserved region of the *Rhg1* locus for detection of copy number variation at *Rhg1* locus (Kadam et al., 2016).

#### Disease resistance

##### Bacterial leaf pustule

Soybean bacterial leaf pustule (BLP) is caused by *Xanthomonas axonopodis pv. glycines*. BLP resistance was reported to be controlled by a single recessive gene (*rxp*) in BLP resistant source, CNS (PI 548445) (Hartwig and Lehman, 1951). QTLs analysis for BLP resistance revealed that Satt372 and Satt486 on Gm17 were strongly associated with BLP resistance (Narvel et al., 2001; Van et al., 2004). Fine mapping in RILs derived from a cross between “Taekwangkong” (BLP susceptible) and “Danbaekkong” (BLP resistant) and two pair of NILs, Kim D. et al. (2010) narrowed down the BLP resistance locus to 33 Kb. Annotation of this 33 Kb sequence identified two putative candidate genes, a membrane protein gene (*Glyma17g09780*) and a Zinc finger family protein gene (*Glyma17g09790*). The candidate genes showed high similarity with their paralogous genes, which were located on the duplicated regions reported for bacterial leaf pustule resistance QTLs (Kim D. et al., 2010). Functional role to these genes has not been assigned.

##### Soybean white mold

White mold, caused by *Sclerotinia sclerotiorum* [(Lib.) W. Phillips.] de Bary, is reported as a devastating disease of soybean and many other crops (Boland and Hall, 1994). Currently, due to the lack of immune type resistance genotypes, only partially resistant cultivars have been used for genetic mapping. Recently, Zhao et al. (2015) identified a major QTL on Gm13 by linkage and association mapping. Four potential candidate genes known to be involved in disease response and anthocyanin biosynthesis were identified at the locus near the peak SNPs. The candidate genes identified were a *PR5-like receptor kinase, RINT-1/TIP-1 family, Myb domain protein 33*, and a *C2H2-type zinc finger family protein* gene (Zhao et al., 2015). These candidate genes will be useful for functional validation and soybean breeding for improving resistance to white mold.

##### Sudden death syndrome

Sudden death syndrome of soybean caused by *Fusarium virguliforme* (Roy et al., 1997), is a serious threat to soybean production (Wrather et al., 2010) which can be managed with host plant resistance (Wen et al., 2014). Resistance to SDS in soybean is multi-geneic and reportedly had two components; a partial resistance to leaf scorch caused indirectly by translocated fungal toxins; and a partial resistance to root infection and rot caused directly at the site of infection by the fungus (Njiti et al., 1998; Kazi et al., 2008). Linkage mapping identified several QTLs associated with SDS resistance across 12 of the 20 soybean chromosomes (http://www.soybase.org/). A locus *Rfs2/Rhg1* on Gm18 underlay partial resistance to the spread of root infections by *F. virguliforme* and SCN (Njiti et al., 1998; Triwitayakorn et al., 2005). Using NILs, Srour et al. (2012) characterized *Rfs2*/*Rhg1* QTL on Gm18 identifying a receptor like kinase (*GmRLK18-1*) responsible for resistance to SDS and SCN. In a GWAS study using two sets of 392 and 300 unique soybean genotypes as independent association panels, Wen et al. (2014) identified a total of 20 loci underlying SDS resistance. Several of SNPs significantly associated with SDS resistance were within or adjacent to sequences annotated as plant disease resistance genes including the previously identified SDS resistance gene *GmRLK18-1*. In a GWAS study using 214 germplasm accessions Zhang et al. (2015b) identified a potential candidate gene on Gm02 encoding *stress induced receptor-like kinase gene 1* (*SIK1*). The peak SNP locus associated with SDS resistance was present in the coding region of the *SIK1* resulting in a non-synonymous mutation.

##### Phytophthora root rot

*Phytophthora* root rot is one of the important diseases of soybean and is caused by *Phytophthora sojae*. Tolerance to *Phytophthora* root rot is multi-genic due to the presence of several races of pathogen and so far 20 *Rps* (resistance to *P. sojae*) loci including 26 alleles have been detected on four different chromosomes i.e., Gm03, Gm13, Gm16, and Gm18 (Li et al., 2016a). Lin et al. (2013) have identified two *Rps* genes, *RpsUN1* on chromosome 3 and *RpsUN2* on chromosome 16 from a soybean landrace PI 567139B, which together confer complete resistance to 16 *P. sojae* races/isolates. Li et al. (2016a), further narrowed *RpsUN1* and *RpsUN2*, to a 151 kb region that harbors five genes including three disease resistance (R)-like genes, and a 36 kb region that contains four genes, all of which are of the typical R-like genes, respectively. RNA-seq analysis of these nine genes before and after inoculation with the pathogen suggest that *Glyma.03g034600* in the *RpsUN1* region, and *Glyma.16g215200* and *Glyma.16g214900* in the *RpsUN2* region of PI 567139B may be associated with the resistance to *P. sojae*. Li et al. (2016b) conducted GWAS in 279 soybean accessions and identified seven candidate genes within 500-kb regions on Gm13 that are probably involved in natural variations in partial resistance to *P. sojae*. These genes encode a COPI (*Glyma13g32980*), a 2OG-Fe(II) protein (*Glyma13g33900*), a PPR protein (*Glyma13g33512*), LRR domain proteins (*Glyma13g33536, Glyma13g33740*), a Gpi16 subunit (*Glyma13g33243*), and a Zn-finger protein (*Glyma13g33260*).

### Genes for abiotic stress

#### Root traits for drought tolerance

Identification of genes underlying root system architecture and canopy characteristics is critical to develop soybean that is suited to water-limited environments. Prince et al. (2015) identified four significant QTLs associated with different root architectural traits on Gm06 and Gm07 in an interspecific RILs population of *G. max* (V71-370) × *G. soja* (PI 407162). A number of candidate genes were identified for root system architecture traits. Notable among them are one encoding triglyceride lipase mapped to QTLs for root distribution traits, two genes encoding an apoptosis inhibitory 5 family protein and an oxidoreductase/transition metal ion binding protein mapped to the taproot length QTLs, a cell cycle associated *D6 type cyclin gene* and a key hormone auxin-associated gene *auxin efflux carrier protein gene* (Prince et al., 2015). In an another study, Manavalan et al. (2015) identified a major QTL on Gm08 that governed root traits (tap root length and lateral root number) and shoot length, and identified six transcription factors (MYB HD, TPR, C2H2 Zn, bZIP, GRAS, and Ring finger) and two key cell wall expansion-related genes which encode xyloglucan endo-transglycosylases as candidate genes in the confidence interval of the QTL. These are key candidate genes for validation and to develop a better root ideotype in soybean.

#### Salt tolerance

Soybean is a moderately salt-sensitive crop and germplasm screening has been shown to display a spectrum of salt-tolerance phenotypes (Munns and Tester, 2008; Phang et al., 2008). A major QTL for salt tolerance has been repeatedly mapped within a region on soybean linkage group N (Gm03) (Lee et al., 2004; Hamwieh and Xu, 2008; Hamwieh et al., 2011; Ha et al., 2013; Guan et al., 2014a). Qi et al. (2014) resolved this QTL in to a single dominant gene *GmCHX1*, a counterpart of *Glyma03g32900* in Williams 82, in a salt tolerant wild soybean accession W05. Comparison of the genomic sequences of W05 and Williams 82 at *GmCHX1* locus encoding a *cation H*^+^*exchanger* revealed that Williams 82 had a ~3.8 Kb *Ty1/copia* retrotransposon inserted into exon 3, but not in its counterpart *Glysoja01g005509* in W05 (Qi et al., 2014). Guan et al. (2014b) resolved the same QTL in to a salt tolerant variety Tiefeng 8, identifying the same gene *Glyma03g32900* (named as *GmSALT3*) with similar insertion of a 3.78-kb *copia* retrotransposon in exon 3 of salt sensitive parent. Haplotype screening of *GmSALT3* identified a total of nine haplotypes including two salt-tolerant haplotypes and seven salt-sensitive haplotypes (Guan et al., 2014b). Recently, Do et al. (2016) identified the same gene in salt tolerant cultivar FT-Abhayra using map-based cloning and identified same insertion of a ~3.8-kb *Ty1/copia* type retrotransposon fragment responsible for loss of function of the salt tolerant gene named as *Ncl*. All of the characterized genes underlying salt tolerant QTLs were pinpointed at single gene *Glyma03g32900*, and the presence of *Ty1/copia* type retrotransposon in all salt tolerant genotypes indicate the single point of origin for salt sensitivity in soybean. Patil et al. (2016) used whole genome re-sequencing data for haplotype analysis of the *GmCHX1* gene (*Glyma03g32900*) identifying three major structural variants and allelic variation in the promoter and genic regions. Based on SNPs specific to three structural variants, breeder friendly KASPar assays have been developed and validated which will be very useful for molecular breeding for salt tolerance in soybean (Patil et al., 2016).

## Prospectus of translational genomics and breeding in soybean

**Figure 2:**
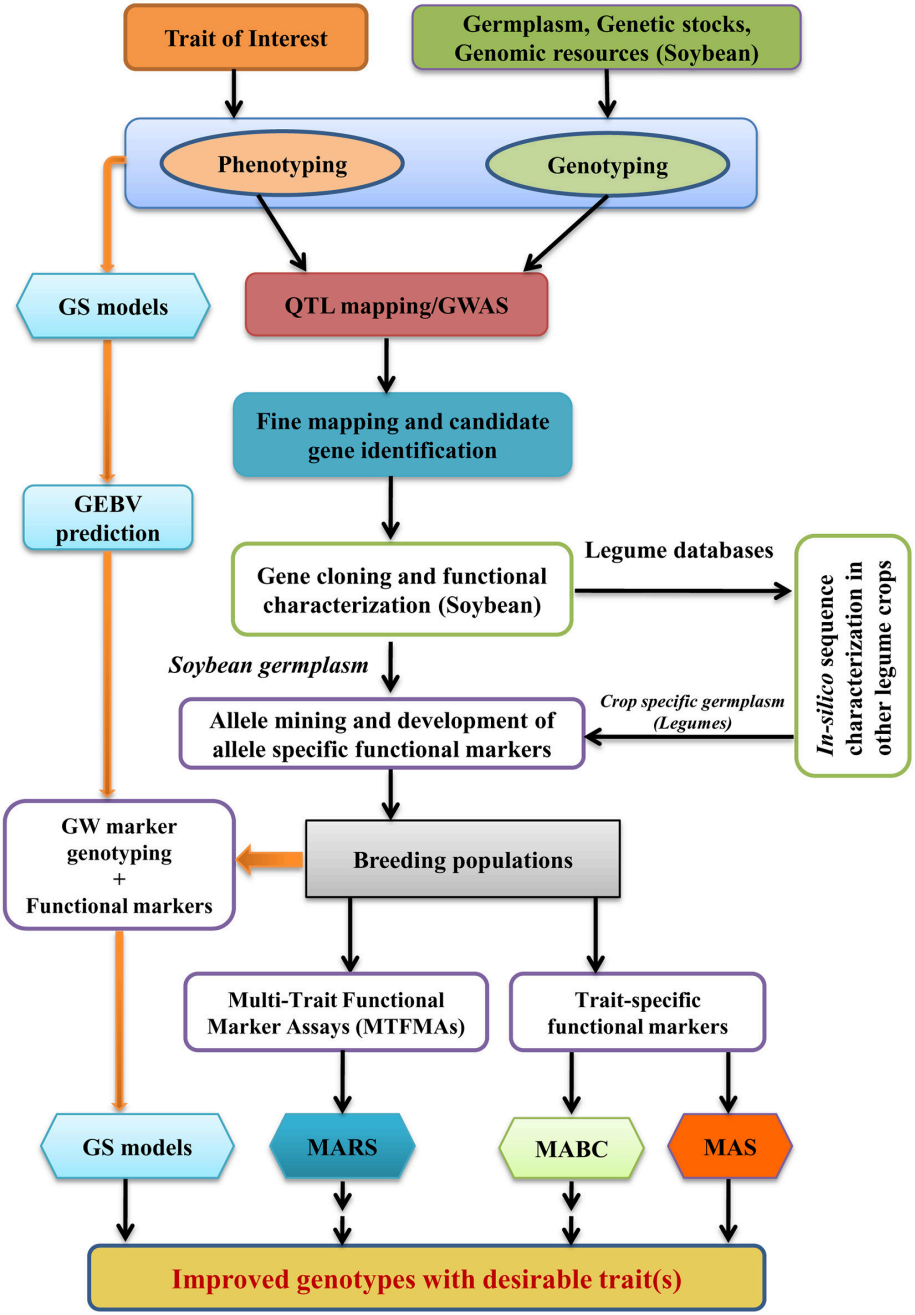
**Strategy for characterization of genes underlying QTLs in soybean and translational genomics enabled breeding in legume crops (GWAS, Genome-wide association studies; MABC, Marker-assisted backcross breeding; MAS, Marker-assisted selection; MARS, Marker-assisted recurrent selection; GS, Genomic selection; GW, Genome wide; GEBV, Genomic estimated breeding values)**.

A lot of phenotypic and genotypic information needs to be generated for QTL mapping, and for identification and characterization of gene underlying a quantitative trait. Once the underlying gene sequence is fully characterized, haplotype analysis of the structural variants identified in the underlying genes for quantitative trait of interest could discover novel and useful alleles leading to functional marker development in soybean. The knowledge of gene function generated through QTL mapping, underlying gene identification, characterization and resulted development of functional markers through allele mining may be translated in to a useful product using genomics assisted breeding approaches, the entire process commonly described as “translational genomics and breeding.” Figure 2 describes the process of translational genomics and breeding in soybean and other legume crops. As soon as functional markers become available, it is much precise and efficient to develop a desirable genotype by using marker-assisted selection (MAS), marker-assisted backcrossing (MABC) and marker-assisted recurrent selection (MARS) compared to traditional breeding methods or linked marker techniques. MAS is useful for selection of individuals with specific alleles for traits controlled by a limited number of loci (upto 6–8). Especially, MAS is effective and time saving for selection of individuals in early generations and in large populations for low heritability traits, allowing breeders to focus attention on a lesser number of high-priority lines in subsequent generations (Collard and Mackill, 2008). MABC method is applied to transfer a limited number of loci (i.e., disease resistance, transgene) from one genetic background to other. In MABC, several cycle of backcrossing is performed to recover the genetic background of the recipient parent (the adapted cultivar) except for the target QTL allele which is selected from donor parent. MABC is very useful for transfer of favorable traits from unadapted germplasm to elite genotypes. However, if the performance of a plant is determined by a complex genotype it is unlikely that this ideal genotype will be attained through MABC only (Ribaut and Ragot, 2006). To overcome the limitation of only being able to improve existing elite genotypes, other approaches like MARS and genomic selection (GS) are considered. MARS involves marker-assisted identification and selection of several genomic regions (up to 20 or even more) for complex traits within a single population (Servin et al., 2004). However, in MARS, major effect QTLs only identified and utilized in selection. These major QTLs can explain only a small fraction of phenotypic variation and may show unexpected trait expression in new genetic backgrounds because of epistatic interactions (Deshmukh et al., 2014).

Another promising approach for genomics assisted breeding of quantitative traits is GS. GS is a form of MAS that selects favorable individuals based on genomic estimated breeding values (GEBVs) of all markers genotyped in a breeding population (Nakaya and Isobe, 2012). These GEBVs say nothing of the function of the underlying genes but they are the ideal selection criterion (Jannink et al., 2010). GS able to capture small-effect QTLs that governs most of the variation including epistatic interaction effects (Deshmukh et al., 2014). However, GS has also got some limitations which are: (1) Each time new GS models has to be trained for new target populations and environment; (2) GS does not provide control over genetic background when a trait is to be incorporated from un-adapted genotype; (3) GS cannot guarantee for selection of a major QTL which is already known. Therefore, it was proposed to incorporate major QTL/GWAS loci information in GS models so that the balance of genetic background can be made along with maximum gain of breeding value (Deshmukh et al., 2014).

Traditionally molecular breeding is performed using linked molecular markers which suffered from limitation of marker polymorphism and genetic recombination, so even if parental genotypes possesses beneficial alleles the selection efficiency may still be <100%. The first example of MAS in soybean was of soybean cyst nematode resistance QTL conditioned by *rhg1* locus (Cregan et al., 1999). The two SSR markers linked with SCN resistance *rhg1* locus were further validated by Silva et al. (2007) and found that only a combination of two markers provide selection efficiency of 100%. Shivakumar et al. (2016) employed MABC for conversion of two high Kunitz trypsin inhibitor (KTI) content soybean varieties (DS 9712 and DS 9814) into low KTI soybean genotypes using three SSR markers. Rosso et al. (2011) has developed breeder friendly markers for *D-myo-inositol 3-phosphate synthase 1* gene (*MIPS1*) which causes modifications to seed phosphorus and carbohydrate content in soybean. In this study, they clearly demonstrated that an allele specific SNP marker developed from causal mutation of *MIPS1* was able to select 100% mutant individuals as compared to four linked polymorphic SSR markers which showed selection efficiency of 42–93%. Therefore, it is necessary to develop functional markers in soybean for selection of QTLs with 100% efficiency. In soybean, functional markers have been developed for several QTLs and a summary of these functional markers is presented in Table 4, which will be very useful for soybean molecular breeders for carrying out translational breeding in soybean. Pham et al. (2010, 2011) used allele specific functional markers for *FAD2-1A* and *FAD2-1B* genes to combine mutant alleles in a single background. They identified soybean lines carrying both homozygous mutant *FAD2-1A* alleles and mutant *FAD2-1B* alleles producing >80% oleic acid content, compared to 20% in conventional soybean. Kumar et al. (2013) successfully used gene specific functional marker for elimination of kunitz trypsin inhibitor from Indian soybean cultivar JS 97-52 through MABC. These are some examples which demonstrate the utility of functional markers developed by characterization of underlying genes of specific traits, to harness the true benefits of translational genomics and breeding in soybean. Although, the functional markers used in the above cited examples were not developed by QTL mapping and characterization, however several functional markers have been recently developed based on characterization of genes underlying QTLs and currently has been utilized in ongoing soybean breeding programs (Tsubokura et al., 2013; Xu et al., 2013; Tardivel et al., 2014; Kadam et al., 2016; Patil et al., 2016).

**Table 4:** **Details of allele-specific functional markers available for translational breeding in soybean**.

**Trait**	**Gene/Marker locus**	**Marker Type (Enzyme)**	**Primer sequences (5′ → 3′)**	**Sizes of amplified fragmengts/restriction digested fragments (bp)**	**SNP/Indel allele**	**References**
Flowering and maturity	*e1-fs/e1-nl*	CAPS (*Hinf I*)	F: CACTCAAATTAAGCCCTTTCA	*E1/e1-as*: 186+36	A/–	Xia et al., 2012
			R: TTCATCTCCTCTTCATTTTTGTTG	*E1-fs*: 136+46 + 36		
				*E1-nl*: No amplification		
	*E1-as*	dCAPS (*Taq I*)	F:TCAGATGAAAGGGAGCAGTGTCAAAAGAAGT	*E1/e1-fs/e1-nl*: 444/443	C/G	Xia et al., 2012
			R: TCCGATCTCATCACCTTTCC	*E1-as*: 413+31		
	*e2*	dCAPS (*Dra I*)	F: AAGCCTATGCCAGCTAGGTATTT	*E2*: 110	A/T	Watanabe et al., 2011
			R: GAAGCCCATCAGAGGCATGTCTTATT	*e2*: 27+83		
	*e3-tr*	FLP	F: TGGAGGGTATTGGATGATGC	*E3-Misuzudaizu*: 1339	–	Watanabe et al., 2009
			R1: CTAAGTCCGCCTCTGGTTTCAG			
			R2: CGGTCAAGAGCCAACATGAG	*E3-Harasoy*: 558		
			R3: GTCCTATACAATTCTTTACGACG	*e3-tr*: 275		
	*e3-fs*	CAPS (*Ale I*)	F: GGGATAGTTCTGATGCTGTTCAA	*E3*: 552+206	–/T	Xu et al., 2013
			R: CCTTGTATCGATAGCATATGTGCT	*e3-fs*: 759		
	*e3-ns*	dCAPS (*Mfe I*)	F: GTTGAAGAGAAGATCACAACA	*E3*: 163	C/T	Xu et al., 2013
			R: GATGAACTAATTTCCCTAACTGCA	*e3*: 140+23		
	*e4-oto*	CAPS (*Sac I*)	F: CCCAGACACTCTTGTGTGAT	*E4*: 535	G/-	Tsubokura et al., 2013
			R: CCATACTCTCGGTATCTTTG	*e4*: 439+96		
	*e4-tsu*	dCAPS (*Eco RV*)	F: CACCCTAGGAGTTGTGTTGTT	*E4*: 355	T/-	Tsubokura et al., 2013
			R: GCGGTTCTGTACAATTGCCTGATA	*e4-tsu*: 332+23		
	*e4-kam*	CAPS (*Afl II*)	F: CTTAATAAAGCCATGACTGGTTTG	*E4*: 494	G/-	Tsubokura et al., 2013
			R: CTTGAGTTTCAATGAGGTTTCAAC	*e4-kam*: 286+208		
	*e4-kes*	CAPS (*BSP HI*)	F: CTTAATAAAGCCATGACTGGTTTG	*E4*: 494	A/-	Tsubokura et al., 2013
			R: CTTGAGTTTCAATGAGGTTTCAAC	*e4-kes*: 399+95		
	*e4-SORE-I*	FLP	F: AGACGTAGTGCTAGGGCTAT	*E4*: 1229	-	Liu et al., 2008
			R1: GCATCTCGCATCACCAGATCA	*e4-SORE-I*: 837		
			R2: GCTCATCCCTTCGAATTCAG			
	*e9*	FLP	F1: GCTCTCTCTCTTCCACTCTCTAGATGG	–	–	Zhao et al., 2016
			F2: ACCCTCTCAAGTGGACATGT			
			R: CTAGGTGCATCGGGATCAAC			
Pod Dehiscence	*Pdh1*	CAPS (*Nhe I*)	F: GCCCTCGTTGTGTTCTTCAT	*Pdh1*: 125	A/T	Funatsuki et al., 2014
			R: GCGTTGCTTCCGTTGTAGAT	*pdh1*: 76+49		
Fragrance	*GmBADH2-A2*	SNAP	F: TGGAAGAAGGTTGCAGACCAGA	Wild type: No amplification	G/A	Juwattanasomran et al., 2011
			R: AAAGCATACCTGCCCTTTACTTTAGAA	Fragrant: 126 bp		
	*GmBADH2-EX10*	Indel	F: TCCCGCCTTATTGTACATGC	Wild type:174	–	Juwattanasomran et al., 2012
			R: TTTTGACCCATTTCACAATCC	Fragrant: 172		
Salt tolerance	M1	KASPar	FAM_primer: ACCAAACCAAACCTAGCTAGTTTTC ATCACCTTCCTATGATTGTTTTTGTTTTAATTTCCT AACTAACTAACACAGCAAG	–	G/C	Patil et al., 2016
			HEX_primer: ACCAAACCAAACCTAGCTAGTTT TCATCACCTTCCTATGATTGTTTTTGTTTTAATTTCC TAACTAACTAACACAGCAAC			
			Common Reverse primer: AAGCACTGAGTCTTTGGCCATGACGTTCAACGCG AGCACCATCACAACGGCGTCGGA			
	M4	KASPar	FAM_primer: TATACAAATATGTTGTTTTGTGT TATTCTACGATTATCATCATCTAGTTC	–	C/G	Patil et al., 2016
			HEX_primer: TATACAAATATGTTGTTTTGTGT TATTCTACGATTATCATCATCTAGTTG			
			Common Reverse primer: AATGGTTAACTTTATCACATATT TATAGTTGAATATAAGTTAGTTT ATTAATTTTCAACTAAAAATTAAC			
	M6	KASPar	FAM_primer: GAGATCTCTTTGTCTCTTCGGGTAACGGCAT TACCAGTTCATTGCTCGTTATACAGGACCGATATTTG	–	G/T	Patil et al., 2016
			HEX_primer: GAGATCTCTTTGTCTCTTCGGGTAACGGCAT TACCAGTTCATTGCTCGTTATACAGGACCGATATTTT			
			Common Reverse primer: ATAAATTCAAACGAAAGTAATCTCGTTAAGACAT CAAGGGCCGAGAGTACTGTGATT AAAGATGCTATCGAAGAACTTTAA			
Soybean cyst nematode	*Rhg1-2*	KASPar	ASP 1: TCTAATGCATTGGTTATAGCAACAACG	–	C/G	Kadam et al., 2016
			ASP 2: TCTAATGCATTGGTTATAGCAACAACC			
			Common primer: TGCTGGCATCTGCCAACTCTGTAAA			
	*Rhg1-5*	KASPar	ASP 1: GAAAGCCAAAGAACTTGAGGAGC	–	C/G	Kadam et al., 2016
			ASP 2: GAAAGCCAAAGAACTTGAGGAGG			
			Common primer: CCAACCACCAGGAATATTAAAGGTACAAT			
	*Rhg4-3*	KASPar	ASP 1: TCGTTGTGTGATTGTTTTGCAGGGA	–	A/T	Kadam et al., 2016
			ASP 2: TCGTTGTGTGATTGTTTTGCAGGGT			
			Common primer: CAGAGATCACAGAGTTTCTCCACCTT			
	*Rhg4-5*	KASPar	ASP 1: GAGGTGGCCGCCGGAGG	–	C/G	Kadam et al., 2016
			ASP 2: GAGGTGGCCGCCGGAGC			
			Common primer: CGACCGCATCATGGGGCTAGAT			
	*Rhg1*	Taqman assay	F: GTTATTACTTCAATCGACGAGTGTGTTG	–	CNV	Kadam et al., 2016
			R:AAATATTTTCCAGTAAAATCAGATTAAAACTATACTTCA			
			FAM: TCGGACACCTCAAAACT			
Oleic acid content	*FAD2-1A*	GC -Tail assay	ASP 1: gcgggcagggcggcATCAACCCATTGGTACTTGC	–	G/A	Dierking and Bilyeu, 2010
			ASP 2: gcgccgATCAACCCATTGGTACTTGT			
			Common forward primer: GTTGCCTTCTCACTGGTG			
	*FAD2-1A*	Simple probe assay	Primer 1: CCAAGGTTGCCTTCTCACTGGT	**-**	A/–	Pham et al., 2011
			Primer 2: TAGGCCACCCTATTGTGAGTGTGAC			
			Fluorescein-SPC-CCTCTAGG**A**AGGGCTGTTTCTCT-Phosphate			
	*FAD2-1B*	Simple probe assay	Primer 1: ACTGCATCGAATAATACAAGCC	–	T/C (In Bold) C/G (Underlined)	Pham et al., 2010
			Primer 2: TGATATTGTCCCGTCCAGC			
			Fuorescence-AGTCCCTTATTTCTCATGGAAAA**T**AAGC–Phosphate			
Raffinose content	*RS2*	GC- Tail assay	ASP 1: gcgggcGTTGCTACCGACCCAGtGAA,	–	C/T	Dierking and Bilyeu, 2010
			ASP 2: gcgggcagggcggcGTTGCTACCGACCCAGcGAG			
			Common forward primer: CAGAGGAATAAAATTCATGAGCATA			
Kunitz trypsin inhibitor	*kti*	PCR	F: CTTTTGTGCCTTCACCACCT	*kti*: 420	–	Alves de Moraes et al., 2006
			R: GAATTCATCATCAGAAACTCTA			

*CAPS, Cleaved amplified polymorphic sequence; dCAPS, derived cleaved amplified polymorphic sequence; FLP, Fragment length polymorphism; CNV, Copy number variation; F, Forward primer; R, Reverse primer; ASP, Allele specific primer*.

## Soybean as model crop for translation genomics and breeding of quantitative traits in legumes

Legumes are quite different from other plant species in certain features such as symbiotic nitrogen fixation, protein rich physiology and secondary metabolism, and past experience has shown that comparative genomics, epitomized by the “model to crop translation” approach, was not very useful for legume crops improvement (Koebner and Varshney, 2006; Varshney et al., 2015). Only few examples of translational genomics are available in grain legumes due to various inherent differences from other non-legume crops (Varshney et al., 2015). Soybean being a legume crop, present a unique opportunity for translational genomics and breeding of quantitative traits in other legume crops, due to presence of legume specific genes and close genetic relatedness to other legume species. In the field of functional genomics, especially in the identification and characterization of genes responsible for quantitative traits, soybean is far ahead from other legume crops. Homology based *in-silico* analysis of the underlying gene sequences (identified from soybean) in other leguminous crops could help in identification of genes for quantitative traits without QTL mapping studies in those legumes. Identification of genetic variants of these genes and functional validation could lead to functional marker development in the particular legume crop (Figure 2). These functional markers can be very useful for molecular breeding in those leguminous crops without spending much money and time for identifying and characterizing QTLs of interest. Although this strategy will not hold true for all of the quantitative traits but will work for many of the legume specific traits. Further, the newly generated reference sequences of unstudied legume species can be annotated by the knowledge of soybean via translational genomics approaches (Kang et al., 2015).

Figure 2 describes the process of translational genomics and breeding in legume crops using genic information from soybean. This process is now very much possible due to the availability of several legume specific databases like Legume Information System (LIS) (Gonzales et al., 2005; Dash et al., 2016), LegumeIP 2.0 (Li J. et al., 2016), SoyBase (Grant et al., 2010) and Soybean knowledge base (SoyKB) (Joshi et al., 2014). The first two databases namely LIS and LegumeIP 2.0 are facilitating translational genomics and breeding in major legumes by integrating genetic, genomic, transcriptomic data, and comparative genomics across important legume crops while the SoyBase and SoyKB are facilitating translational genomics and breeding mainly in soybean. Zhang X. et al. (2016) demonstrated the utility of legume specific comparative genomics through *in-silico* analysis and ectopic expression of *E1*, a key flowering regulatory gene in soybean. Flowering regulation pathway in soybean is quite distinct from that identified in model plants (Jung et al., 2012). Comparative genomic analysis of soybean flowering genes using Arabidopsis genome annotation as a model didn't detect *E1* gene until Xia et al. (2012) cloned and characterized it (Jung et al., 2012). However, Zhang X. et al. (2016) was able to identify a total of nine *E1* family genes from six legume species, using soybean *E1* sequence in an *in-silico* analysis. Functional conservation and diversification was observed among *E1* family homologs of the six legumes (Zhang X. et al., 2016). Identification of allelic variants and haplotype analysis of *E1* family genes among legume crop specific germplasm will help in the development of breeder friendly molecular markers.

Translational researches enabled discovery of large number of causative SNPs and InDel alleles for multiple traits opens window for designing crop specific Multi-Trait Functional Marker Assays (MTFMAs), which can be used for screening large breeding populations. These MTFMAs will help in combining several beneficial alleles from multiple parents through MARS in multi-parent breeding populations (Figure 2). MTFMAs will also reduce the cost and time of phenotyping large breeding populations and help the breeders of resource poor countries to develop tailor-made varieties of legume crops to suite their specific needs.

## Difficulties, challenges, and potential errors in QTLomics and translational genomics

Several factors influence QTL detection in a mapping population i.e., the QTL allele effect, the existence of other linked QTL, the mapping population size, extent of genetic recombination, phenotypic assessment accuracy, genotyping errors, missing data, and environmental effects (Collard et al., 2005; Francia et al., 2005). Further, independent populations chosen from different gene pools may express different phenotypic values in different environments. Another difficulty for translational genomics is un-expected expression of introgressed QTLs in a new genetic background. The genomic region associated with a QTL may hold not just one but several genes, and recombination between those genes would then modify the effect of the introgressed segments. Since, biochemical pathways and enzyme protein sequences are known for some of seed composition traits, candidate gene identification is much easier for these traits resulting in the development of several functional markers in soybean i.e., oleic acid content, isoflavone content, and raffinose content etc. (Dierking and Bilyeu, 2010; Pham et al., 2010, 2011; Wang et al., 2014). However, for agronomic and yield related traits, biochemical pathways are not well-characterized and therefore QTL mapping is the major strategy for identification of underlying genes. Although several genes are involved in the biological network of a quantitative trait, we are only able to detect the variation present in the parental genotypes and gene pool utilized for QTL mapping. This is further complicated by epistatic gene interactions and the target phenotypic environment. So, the major challenge in soybean QTLomics is to capture the genetic variation present among whole soybean gene pool for specific quantitative trait in target environment. The recent advances in NGS technologies and high-throughput marker genotyping enabled genotyping of whole germplasm accessions allowing GWAS for mapping of QTLs in whole gene pool. The large data-sets generated from NGS and high density genotyping requires sound computational algorithms for detection of minor QTLs as well as rare alleles with major effect phenotype. The biggest challenge for QTLomics is to integrate data from genome sequencing, transcriptomics, the epigenome and phenotyping, to make sense of it all. To overcome genetic background effect functional markers used for MAS can be combined with GS models (Deshmukh et al., 2014). The major challenges for translational genomics in legumes includes: (1) limited availability of predictive and functional markers for target traits, (2) limited access to high-throughput genotyping technologies, (3) limited availability of high-throughput, cost-effective and precise phenotyping platforms, (4) availability of automated data management, analyses, and interpretation systems (Varshney et al., 2012, 2015). Genotyping errors, false discovery rate, over-estimation of QTL effect and non-detection of superior but rare alleles are potential errors which QTLomics and translational genomics frequently encounter.

## Conclusion and future perspectives

The identification of genes underlying complex traits in soybean is gaining momentum and a number of genes/candidate genes have been identified for many quantitative traits. The discovery of causative alleles for a number of quantitative traits in soybean could be used to develop functional markers for combining multiple traits from several genotypes in a cost-effective and efficient way. Already several functional markers have been developed and validated for many quantitative traits to facilitate translational research enabled breeding in soybean. This will certainly help in the development of ideal plant types of soybean to suit the specific requirements, besides facilitating molecular breeding in other legume crops via comparative genomics. However, genes for many quantitative traits of economic interest i.e., number of pods, oil content, photosynthetic efficiency, nitrogen use efficiency, thermo-tolerance etc., which are the most desirable genes in soybean breeding are yet to be identified. The potential for identifying genes for these traits lies in the approaches like next-generation sequencing based mapping in multi-parent cross derived mapping populations, mapping in advanced inter-crossed line mapping populations, association mapping using high throughput genotyping and phenotyping technologies, pooled sequencing based approaches, and targeted genome editing methods, which needs to be taken up at high priority by various soybean genomics research groups and by an International consortium on soybean functional genomics.

## Author contributions

GK planned the MS content, coordinated with other coauthors, contributed in drafting special sections, and finalizing the manuscript. SG, MR, SM, and GS contributed specific sections of this manuscript. SG was involved in planning the content, editing, and finalizing the manuscript.

### Conflict of interest statement

The authors declare that the research was conducted in the absence of any commercial or financial relationships that could be construed as a potential conflict of interest.
